# Optimization of lactic acid fermentation for pathogen inactivation in fecal sludge

**DOI:** 10.1016/j.ecoenv.2018.03.075

**Published:** 2018-08-15

**Authors:** Emmanuel Alepu Odey, Zifu Li, Xiaoqin Zhou, Yichang Yan

**Affiliations:** School of Energy and Environmental Engineering, Beijing Key Laboratory of Resource-Oriented Treatment of Industrial Pollutants, University of Science and Technology Beijing, Xueyuan 30, Beijing 100083, PR China

**Keywords:** Fecal sludge, Fecal coliform, Lactic acid fermentation, Lactobacillus, Fermented cassava flour, Fermented rice flour

## Abstract

The efficiency of lactic acid fermentation (LAF) as a pretreatment for human feces was investigated in laboratory-scale experiments that lasted for 3 weeks. The sanitization effect of LAF on fecal sludge (FS) was conducted in triplicate. This study used three materials, namely, lactobacillus of lactic acid bacteria, fermented cassava flour, and fermented rice flour, which were known to enhance the production of lactic acid. Each material was mixed in three different reactors at equal ratio with raw FS (i.e., 1:1 v/w, w/w, and w/w). The pH decline rate, lactic acid production rate, and fecal coliform suppression degree were monitored over the period of the treatment process as parameters to evaluate the efficiency of various LAF for pathogen inactivation in FS. Results showed that only fermented rice flour was able to completely inactivate the indicator organism (fecal *coliform*) at the end of fermentation. Final plate counts of 8.6 × 10^8^ CFU/100 mL, 2.4 × 10^8^ CFU/100 mL, and zero (0) were achieved from lactobacillus, fermented cassava flour, and fermented rice flour treatment processes, respectively. The final pH from the reactors that contained lactobacillus and FS, cassava flour and FS, and fermented rice flour and FS were 5.5, 8, and 3.9, respectively. This study revealed that not all LAF materials can effectively suppress pathogens in FS. The results serve as the foundation in developing an effective, cheap, and easy to use LAF on FS pretreatment for pathogen inactivation.

## Highlights

• Lactobacillus, fermented cassava flour and fermented rice flour were used to produce lactic acid for pathogens inactivation in fecal sludge.
• Fecal coliforms were completely inactivated within 3 weeks of rice flour fermentation with fecal sludge.
• No significant decline in fecal coliform during lactobacillus and fermented cassava fermentation.
• Fecal pH was permanently reduced during fermentation with rice flour.

## Graphical abstract

**Figure:**
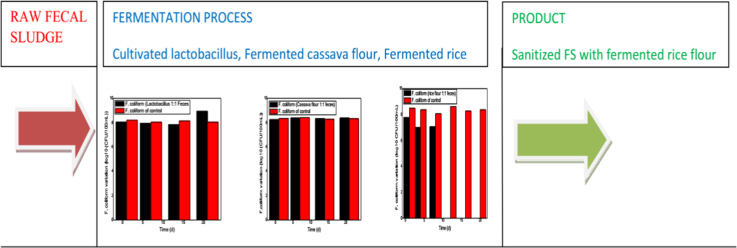

## 1 Introduction

Adequate facilities for the safe treatment or disposal of fecal sludge (FS) should be established globally. FS causes several harmful diseases if not properly managed (al-Ghazali and al-Azawi, 1990; Bracken and Ysunza, 2005; Jiang et al., 2002). Approximately 2.5 billion people globally do not have access to adequate sanitation facilities (UN, 2012), and more than 0.7 billion people globally lacked access to clean water because the water is polluted by people's own feces (UN, 2012). A study revealed that about 1800 children die every day due to diseases associated with inadequate hygiene, lack of sanitation, and contaminated water (UNICEF, 2013).

To address these issues, the millennium development goal (MDG) target aimed to reduce the 2.5 billion people without adequate sanitation in half by 2015. Unfortunately, this target was not feasible at the end of 2015. Considering that the world's population is expected to reach 9.6 billion in 2050, a suitable method for dealing with FS, especially on the processes that enable the sustainable and efficient recovery of resources, should be developed (UN, 2012, Yemaneh, 2015). In the past few years, dry and low water sanitation has gained worldwide attention with the concept that FS can be directly processed at the point of recovery or collected and transported to the point of treatment (Mackie Jensen et al., 2008, Verbyla et al., 2016, Yemaneh et al., 2012). Several products, such as fertilizers, biofuels, water, biogas and compost, can be recovered after treatment (Bracken and Ysunza, 2005; Jepsen et al., 1997; World Health Organization (WHO) (WHO) and (UNICEF), 2014).

Several methods have been introduced to successfully treat FS for hygienic end-product for re-use or disposal (Allievi et al., 1994, Anderson et al., 2015a, Factura et al., 2011; Feachem, 1983; Magri et al., 2015). Lactic acid fermentation (LAF) has been reported to successfully suppressed pathogens in FS and organic material preservation (Anderson et al., 2015b). LAF is a metabolic process in which lactic acid fermenting organisms (LAB) easily metabolize degradable carbohydrates to lactic acid. LAB have the capability to convert carbohydrates to lactic acid, and the genera *Leuconostic*, *Lactobacillus*, and *Streptococcus* are used for food preservation by fermentation industries and sanitation agencies (Vandenbergh, 1993). The reduction of pH during lactic acid fermentation and the production of antimicrobial compounds are effective natural and cheap processes to effectively eliminate non-desirable microorganisms and pathogens when applied for hygienization (Noike et al., 2002). Lactic acid reduces the bulk pH of the surrounding medium that influences the activity of membrane-bound enzymes and exo-enzymes (Anderson et al., 2015b). In addition, the capability of lactic acid to suppress pathogens is partially attributed to its capability to penetrate the cytoplasmic membrane of microorganisms in the associated form, which results in the decline on intracellular pH of pathogens. Zhu et al. (2006) reported that the survival of pathogens pH was reduced at pH less than 3.5 although the suppression of bacteria requires a pH of less than 2.5. Therefore, the key antimicrobial property of lactic acid is the capability to suppress the intracellular of bacteria (Anderson et al., 2015b). Scheinemann et al. (2015) reported that bacteria pathogens, such as *E. coli, salmonella, and Staphylococcus aureus* were eliminated from cow manure after few days of fermentation. Anderson et al. (2015b) also reported that fecal *coliform and E.* coli were reduced below detection limit within 1 week of FS treatment with LAB created through the mixture of fermented milk that contained *lactobacillus casei* and pasteurized whole milk.

Although several studies have been conducted to investigate the sanitizing potential of lactic acid within the food industry (Anderson et al., 2015b, Mante et al., 2003), studies that focused on the sanitizing potential of lactic acid on pathogens present in FS are limited. In addition, no concrete studies have been conducted to compare the efficiency of LAF of FS. An experiment was conducted in this study to compare the LAF treatment efficiency of FS with LAF materials, namely, lactobacillus strain of LAB, fermented cassava flour, and fermented rice flour. The results served as the foundation for the research on producing effective, cheap and easy to use LAF for safe application or disposal of FS hygienization.

## 2 Materials and method

### 2.1 Origin of fecal sludge and experimental set-up

FS was collected from the septic tank of the University of Science and Technology, Beijing. The initial characteristics are shown in Table 1. FS was collected from the septic tank at the same day of fermentation with the prepared lactic acids to avoid the changes in microbial communities and pH variation. FS was transported to the laboratory in a 10 L bucket. In the laboratory, a suction pump was used to fill each of the four 5 L buckets with approximately 1 kg for control bucket, 1 kg for bucket marked with 1:1 of FS and lactobacillus, 1 kg for the bucket with 1:1 of FS and fermented cassava flour, and 1 kg for bucket marked with 1:1 of FS and fermented rice flour. Each of the buckets was designated as reactors 1–4. Prior to the commencement of the stabilization experiments, 50 g of raw FS was used for the analysis on physical and microbial properties. The indicator organism, fecal coliform, was used to assess the overall sanitation efficiency of various fermentations. Serial dilution of the fecal sample was conducted in deionized water for microbial test. Fecal coliform count was determined using a membrane method with coliform agar, followed by incubation at 48 °C for 24 h.

**Table 1:** Initial fecal sludge characteristics.

	Unit	Initial fecal sludge characteristics		
Parameters				Values
Temperature	°C			21
pH				7.53
Total solid	%			14.41
Fecal coliform	CFU/100 mL			3.1 × 10^8^

## 3 LAF

**Fig. 1:**
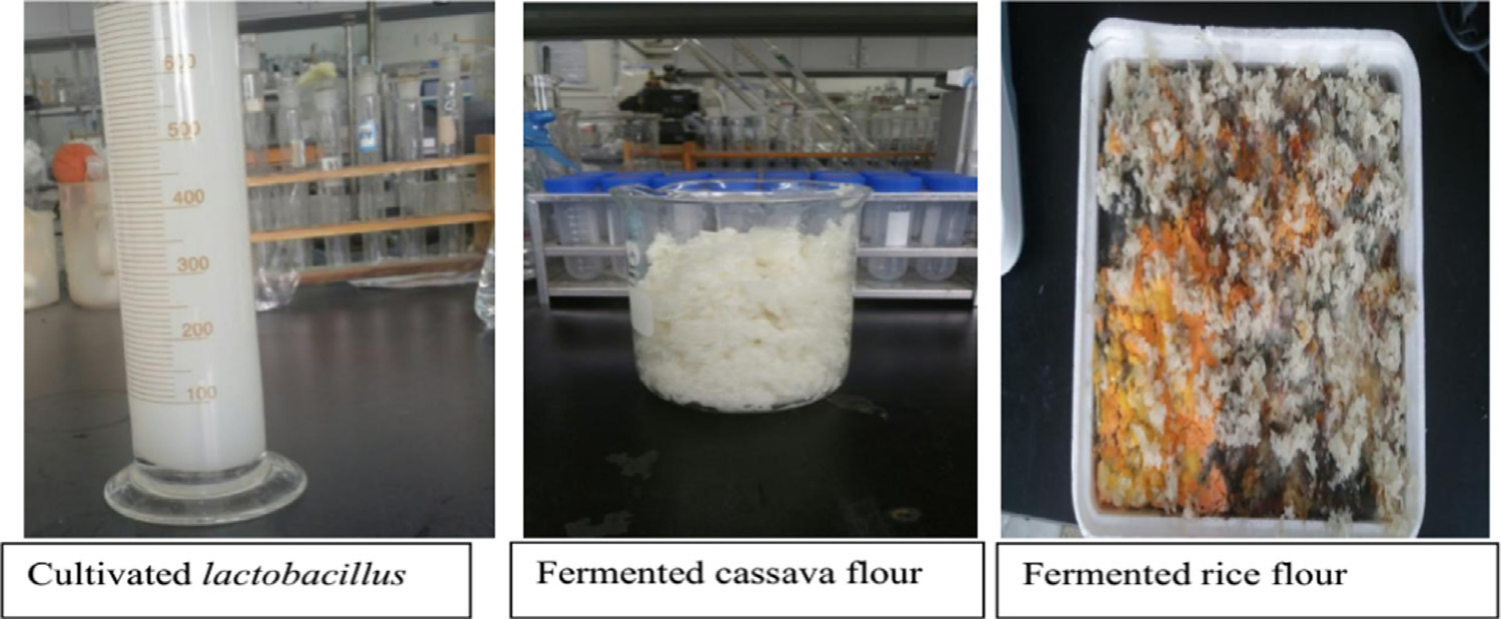
Cultivated lactobacillus, fermented cassava flour and fermented rice flour.

The current approach for lactic acid production uses food waste and agricultural products. The three materials used were distinguished into different materials. *Lactobacillus* strain and cassava were classified as starch materials, and rice was classified as cellulose products. All the three fermentations lasted for 6 d. The initial volume of lactobacillus used was 40 mL, cultured every 24 h, and was enlarged to the required volume of 1 L with the addition of deionized water for the period of 6 d at room temperature for the LAB to grow to a large quantity before mixing with FS for the treatment process. For the fermentation in cassava flour, dry cassava flour was soaked with deionized water and was allowed to ferment for a period of 6 d. The main stages involved in the lactic acid production from fermented rice flour included steaming, soaking, extruding, boiling, cooling, and fermenting. Soaking is important for the production of fermented rice flour because it increases the water content of rice and enhances natural fermentation. pH values of 4.2, 3.8, and 3.4 were achieved from *lactobacillus*, fermented cassava flour, and fermented rice flour on the 6th day of fermentation. Fig. 1 shows the LAF process of lactobacillus, fermented cassava flour, and fermented rice flour.

### 3.1 Analytical method

The total solid content of initial FS and after treatment were assessed using the 2540E standard method for wastewater examination. The LAF treatment process was monitored by measuring the concentration and accumulation of lactic acid bacteria and pH variation. The use of lactic acid for the inactivation of fecal coliform was conducted by evaluating the survival rates of pathogens through a total viable plate count method and pH changes during the treatment process. LAB was cultured using a pour plate technique on lactobacillus select agar and were incubated at 37 °C for 24 h. The total viable counts of bacteria colonies on solidified agar plates using chromogenic agar technique were used to measure the level of fecal coliform suppression. The fecal coliform count was determined using the membrane method with coliform agar, followed by incubation at 48 °C for 24 h. The treatment period was set as the time required for fecal coliforms to appear on the agar. The number of fecal coliforms (CFU/100 mL) was determined for each analyzed sample by using Chromocult® Coliform agar. The culture media was prepared using standardized protocols and reagents under sterile conditions. This process was conducted in an electric oven operated aerobically. The pH values during the treatment processes were determined by taking 10 g of sample from each reactor that was dissolved in 100 mL distilled water. The dissolved portions were stirred for 15 min. After settling, the liquid portion was measured through potentiometric measurement using standard pH electrode.

## 4 Results and discussion

### 4.1 Lactic acid production

To determine the lactic acid content and production efficiency of LAF from fermented materials and their fermentation efficiency when applied in LAF of FS, the community composition and dynamics of LAB associated with *lactobacillus*, cassava flour, and rice flour fermentation were investigated by using a culture-dependent approach. Single strain of the fermentation materials (i.e., *Lactobacillus fermentum*, *Lactobacillus amylovorus*, and *Lactobacillus Sp.*) was isolated. The results showed that *Lactobacillus Sp.,* which was a strain from fermented rice flour, produced high lactic acid content compared to cassava flour and *lactobacillus*. Furthermore, the lactic acid production efficiency of fermented rice flour was higher than that of cassava flour and *lactobacillus*, as shown in Table 2. This result revealed that fermented rice flour can be an effective substrate for LAF of FS for pathogen reduction and odor control due to its efficiency in producing high amounts of lactic acid during fermentation. A comprehensive analysis on fermentation aids in devising strategies to improve the quality of LAF of FS.

**Table 2:** selected bacteria of *Lactobacillus* strain and their lactic acid production in culture media with the three different substrates.

Medium	LAB strain	Type of LAF	Lactic acid content g/L	Production efficiency of lactic acid g/L h^−1^
Lactose	Lactobacillus fermentum	Heterofermentation	15.0	2
Cassava flour	Lactobacillus amylovorus	Heterofermentation	4.8	0.2
Rice flour	Lactobacillus Sp.	Homofermentation	129.0	2.9

## 5 Comparison treatment processes

**Fig. 2:**
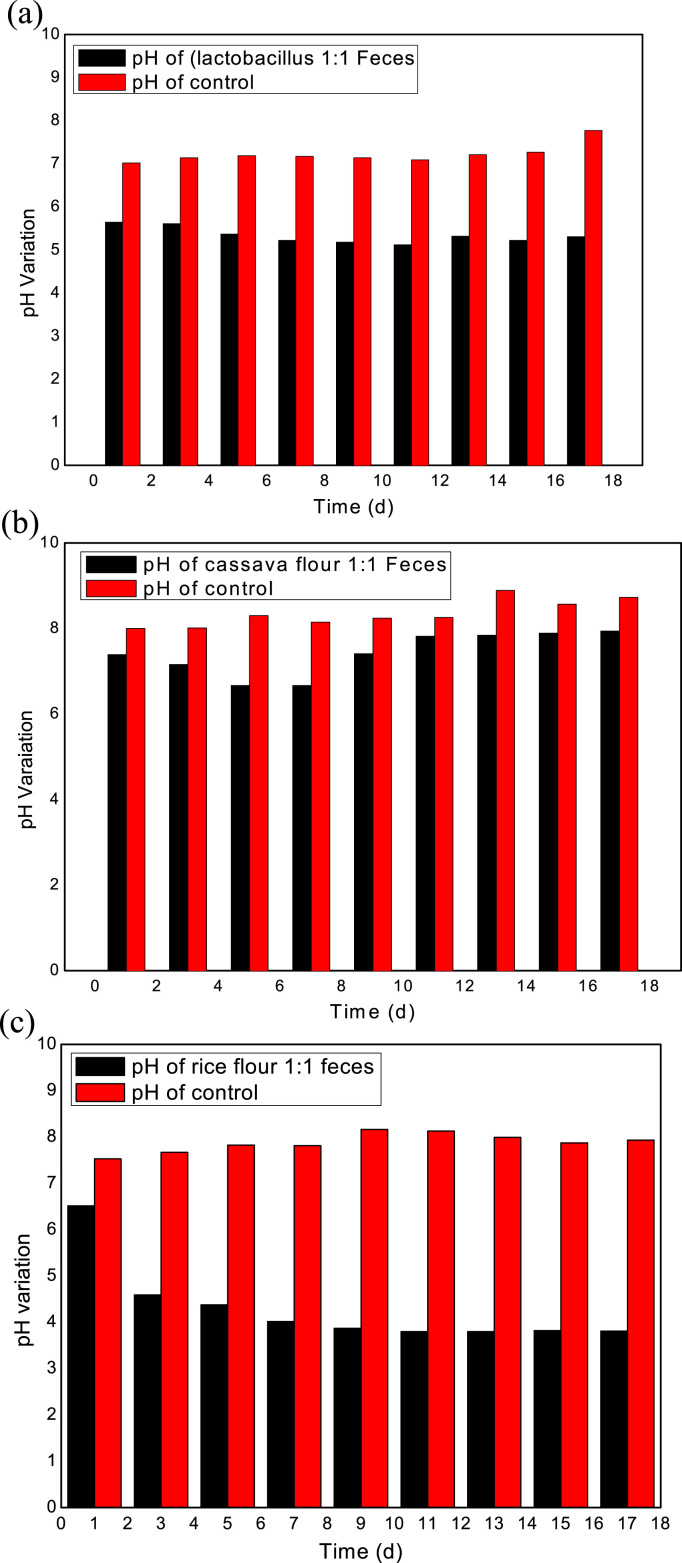
(a) pH variation during FS fermentation process with the cultivated *lactobacillus*. (b) pH variation during FS fermentation process with fermented cassava flour. (c) pH variation during FS fermentation process with fermented rice flour.

pH variation in the treatment process is an important parameter that should be considered in LAF experiments because it determines the acidity and alkalinity of fermentation (Anderson et al., 2015a, Factura et al., 2011; Feachem, 1983). pH was used in this experiment to evaluate and compare the fermentation efficiency from various treatment processes. Result showed that the single strain of LAB (*lactobacillus*) was able to reduce the pH of treatment processes to 5.8 when initially mixed with FS in equal ratio and declined to 5.2–5.4 throughout the period of the experiment, and the control pH remained at nearly the same level, as shown in Fig. 2a. This study revealed that a single strain of LAB cannot effectively suppress pH variation in FS. The acidity of FS influences the rate of change in pH, which determines the amount of lactic acid present in fermentation and the effective sanitization. Thiamann (1963) stated that for lactic acid fermentation to be effective in sanitization, a final pH of approximately 4.0 should be achieved. However, this study showed that effective sanitation process depended on the initial low pH value of lactic acid and the survival rate of LAB when added to FS. The initial pH value of the cultivated *lactobacillus* was 4.2 before adding to FS. However, the process did not suppress the pH to below 5.4.

In the reactor that contained fermented cassava flour and feces, the pH value reduced to 7.5 few hours after mixture with FS and was reduced to 6.0 on the 5th and 7th day. The pH value of the system started increasing on the 10th day and remained high throughout the remaining period of the experiment, as shown in Fig. 2b. A final pH of 7.8 was observed. According to Sanni et al. (2013), fermented cassava produced LAB, such as *Pediococcus, Lactobacillus fermentum*, and *Lactobacillus.* However, no significant decline in pH toward acidification was observed when fermented cassava was applied for the sanitization of FS in this study. This finding may be due to the lack of supplement sugar source to keep the produced LAB active during the treatment process. Kamra and Srivastava (1994) reported that the application of LAF for fermentable substances requires sugar supplements. FS does not contain easily degradable sugar, and easily degradable sugar supplements are required to enhance the growth of LAB during fermentation with LAF materials (Lopez Zavala et al., 2004).

In the third reactor that contained fermented rice flour and FS, the pH value reduced to 6.6 on the first day of mixture and reduced to 3.9 on the 7th day of fermentation. The pH value stabilized from the 10th day to the final day of treatment with the pH value that ranged between 3.7 and 3.9, as shown in Fig. 2c. The decline and stability in pH showed that fermented rice flour can produce LAB capable of suppressing pathogens. This finding implied that fermented rice flour could be an effective lactic acid conditioner for pathogen inactivation in FS because pathogens, such as *Staphylococcus aureus, coliform* and *Salmonella spp*., rarely survive in acidic environment with low pH (Glaser and Guggenberger, 2001). pH reduction is an important aspect of hygienization and preservation-oriented LAF process (UNICEF, 2013). In this study, only reactor mixed with rice flour and feces was able to reduce pH of the treatment process to the suitable acidification range.

## 6 Inactivation of fecal *coliform*

**Fig. 3:**
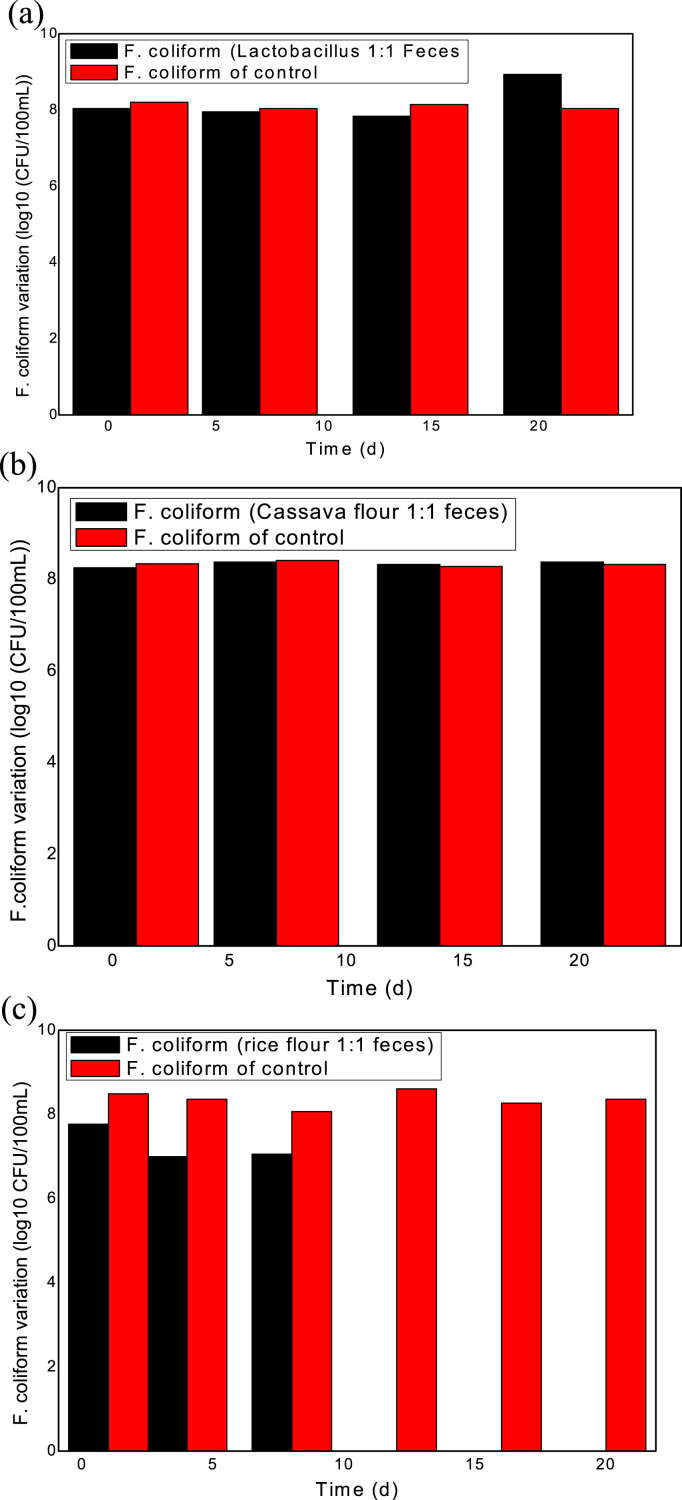
(a) Fecal coliform variation in the reactor containing lactobacillus and FS. (b) fecal coliform variation in the reactor containing fermented cassava flour and FS. (C) fecal coliform variation in the reactor containing fermented rice flour and FS.

On the basis of previous studies, assessing the efficiency of treatment processes in FS fermentation is expensive and requires rigorous labor to measure all types of available pathogens (Strande et al., 2014). Thus, an indicator organism should be selected to measure the pathogenic activity during fermentation to indicate the amount of eliminated pathogens during the entire treatment process (Chahal et al., 2016; Feachem, 1983; Strande et al., 2014). Fecal *coliform* was used in this experiment as the only indicator organism to evaluate and compare the treatment efficiency of produced lactobacillus in the laboratory, fermented cassava flour, and fermented rice flour for pathogen inactivation in FS. The results showed that *lactobacillus* was able to slightly reduce fecal *coliform* concentration to 1.1 × 10^8^ CFU/100 mL after 5 h of fermentation with FS, and fecal *coliform* count of 1.6 × 10^8^ CFU/100 mL was recorded from the control reactor at the same time. Interestingly, fecal coliform count at the final day of treatment in the fermentation reactor was more than the one recorded from the control reactor. Final fecal *coliform* of 8.6 × 10^8^ CFU/100 mL was recorded from the fermentation, and 1.16 × 10^8^ CFU/100 mL was recorded from the control reactor. The results are calculated and converted to log10^8^ CFU/100 mL and are presented in Fig. 3a. This result revealed that lactobacillus only cannot completely eliminate the pathogens in FS.

Results from the reactor that contained cassava flour and FS showed that the process was only able to reduce fecal *coliform* content to 1.8 × 10^8^ CFU/100 mL within 5 h of treatment compared to 2.2 × 10^8^ CFU/100 mL observed from the control reactor. Fecal *coliform* of 2.4 × 10^8^ CFU/100 mL was achieved on the final day of treatment. The results are converted to log10 CFU/100 mL and are presented in Fig. 3b. The incapability of fermented cassava flour to completely inactivate bacteria pathogens in the FS may be due to the incapability of the process to reduce the pH of the system to acidic state.

The sanitation indicator bacteria were reduced to 0.6 × 10^8^ CFU/100 mL in the reactor that contained fermented rice flour and feces within 1st 5 h period with the indicator reactor that had fecal coliform count of 3.1 × 10^8^ CFU/100 mL. The sanitation indicator bacteria were completely eliminated on the 15th day of fermentation. The results are converted to log10 CFU/100 mL and are presented in Fig. 3c. The total elimination of fecal *coliform* in this process may be attributed to the low pH value achieved from the process because a previous study reported that LAF process that achieved a final pH value of 3.5–4.2 efficiently eliminated a range of pathogens. The final pH achieved in the fermentation with rice flour was within 3.7–3.9. Apart from the stable reduction in pH, other factors may have played a vital role in fermented rice flour that contributed in eliminating bacteria pathogens in the FS. Li et al. (2015) reported that fermented rice flour produces abundant LAB and yeast. This condition may contribute in suppressing bacteria pathogens in FS. However, other antimicrobial compounds, such as hydrogen peroxide, diacetyl, and bacteriocins are reported to be produced by LAB during fermentation, which also play important roles on pathogen elimination in the fermentation system (Mackie Jensen et al., 2008, Verbyla et al., 2016, Yemaneh et al., 2012).

## 7 Hygienization of bacteria pathogen

**Fig. 4:**
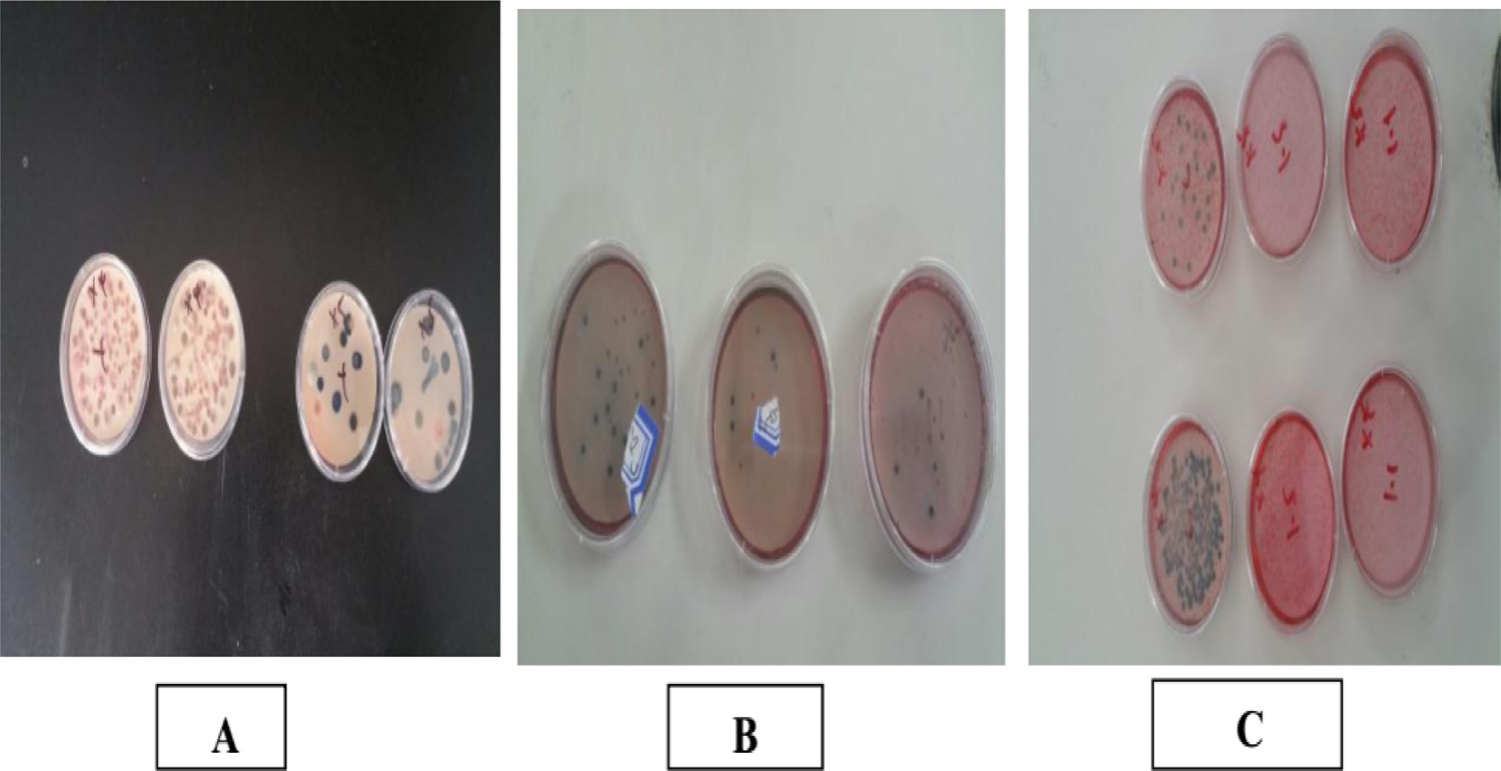
Fecal coliform plate count after final treatment (A= Treatment with lactobacillus, B= Treatment with fermented cassava flour, C= Treatment with rice flour), the plates on the left in each picture show control plates, while the plates on the right show treatment plates.

Considering that LAB in fermented rice flour remained active throughout the experiment, the hygienization method showed more effectiveness than fermented cassava flour and *lactobacillus* bacteria because fecal *coliform* was reduced to half compared with the control reactor within one week and were completely inactivated within two weeks. The final plate count from the treatment processes is presented in Fig. 4. The method used in this study reduced fecal *coliform* more efficiently than the lime treatment, ammonia treatment, and composting process. Liu (2016) confirmed that *enterococci* and *coliform* were reduced during composting within a period of months, but traces of residual concentrations were still observed in the mature compost. This method conserved the nutrients during the processing of FS effectively due to the absence of self-heating technique and the minimal production of gas. Food supply through agriculture should be improved by using sanitized FS as soil conditioner in the growing global population. However, the direct use of lacto-fermented feces to agriculture may be constrained by high concentrations of organic acids, insufficient hygienization, and incomplete decomposition. Post-treatment by vermin-composting, thermophilic composting and the addition of biochar stabilize and sanitize the material before its application in agriculture.

## 8 Odor evaluation

Odor evaluation by panel of observers indicates that the fecal odor is not suppressed from the treatment process and the control for experiments with *lactobacillus* and FS. For the experiments with the mixture of fermented cassava flour and FS (1:1 W/W), fecal odor is suppressed and replaced by cassava flour smell. For the treatment with the combination of fermented rice flour with FS (1:1 W/W), the fecal odor is suppressed and is replaced by sour smell. From the results of different monitored processes for LAF experiments of *lactobacillus*, fermented cassava flour, and fermented rice flour, the LAF of rice flour indicates better odor suppression efficiency compared with other LAF sources used in the study. Therefore, fermented rice flour played an important role in odor control for effective LAF of FS.

## 9 Conclusion

In this study, the treatment efficiency of LAF processes for pathogen inactivation in FS was conducted among LAB potential materials, namely, *lactobacillus*, fermented cassava flour, and fermented rice flour. The results showed that only fermented rice flour effectively inactivated the indicator organism (fecal coliform). The final pH values of 5.4, 7.8, and 3.9 were achieved from *lactobacillus*, fermented cassava flour, and fermented rice flour, respectively. The results indicated that acidification during fermentation played a vital role in pathogen elimination, because only rice flour was able to reduce the pH of the process toward acidification state. This study revealed that not all LAF materials had sufficient native lactic acid fermenting microorganisms that can effectively suppress bacteria pathogens in FS and several LAB potential materials required additives, such as sugar, to keep the process active. Therefore, proper pretreatment test should be conducted to select a suitable method for LAF on the effective acidification of FS for pathogen elimination.
